# Clinical Efficacy and Safety of Shenqu Xiaoshi Oral Liquid for Functional Constipation in Children: A Systematic Review and Meta-Analysis

**DOI:** 10.3390/children13040464

**Published:** 2026-03-27

**Authors:** Shen Li, Haobo Xu, Tian Geng, Zhongyue Li

**Affiliations:** 1 Department of Pediatrics, The Fourth Affiliated Hospital of School of Medicine, and International School of Medicine, International Institutes of Medicine, Zhejiang University, Yiwu 322000, China; 22518620@zju.edu.cn (S.L.);; 2 Department of Pulmonology, Children’s Hospital, Zhejiang University School of Medicine, National Clinical Research Center for Child Health, Hangzhou 310052, China

**Keywords:** Shenqu Xiaoshi Oral Liquid, functional constipation, children, meta-analysis

## Abstract

**Aim:** We aimed to evaluate the clinical efficacy and safety of Shenqu Xiaoshi Oral Liquid in the treatment of functional constipation in children. **Methods:** A comprehensive literature search was conducted from inception to 20 October 2025, across PubMed, Embase, Scopus, Web of Science, the Cochrane Library, Chinese VIP Information Database, China National Knowledge Infrastructure (CNKI), and Wan Fang Med Database. For quantitative analysis, the mean difference (MD) was used for continuous outcomes and the risk ratio (RR) for dichotomous outcomes. The methodological quality of the included studies was assessed using the Cochrane risk of bias tool. Statistical analyses were performed using RevMan 5.3 and Stata 13 software. **Results:** Eight studies involving 692 pediatric patients were included (Shenqu Xiaoshi Oral Liquid group: 345; control group: 347). Compared to the control group, Shenqu Xiaoshi Oral Liquid demonstrated superior clinical effectiveness [RR = 1.36, 95% CI: (1.25, 1.47); z = 7.11, *p* < 0.00001] and a lower recurrence rate [RR = 0.49, 95% CI: (0.26, 0.93); z = 2.18, *p* = 0.03]. Both the post-treatment [WMD = −0.91, 95% CI: (−0.97, −0.86); z = 31.94, *p* < 0.00001] and post-recurrence [WMD = −1.49, 95% CI: (−1.56, −1.41); z = 40.12, *p* < 0.00001] defecation intervals were shorter in the Shenqu Xiaoshi Oral Liquid group. No significant difference was observed in the incidence of adverse reactions between the two groups [RR = 0.67, 95% CI: (0.35, 1.29); z = 1.20, *p* = 0.23]. Furthermore, serum levels of motilin [WMD = 41.66, 95% CI: (34.17, 49.16); z = 10.90, *p* < 0.00001] and gastrin [WMD = 23.74, 95% CI: (7.30, 40.19); z = 2.83, *p* = 0.005] were significantly higher in the Shenqu Xiaoshi Oral Liquid group. **Conclusions:** Shenqu Xiaoshi Oral Liquid shows favorable clinical efficacy and an acceptable safety profile for treating functional constipation in children. However, these outcome measures are influenced by the limited sample size and potential heterogeneity of the included studies, warranting cautious interpretation of the results.

## 1. Introduction

With socioeconomic development and changes in dietary structure and lifestyle habits, functional constipation (FC) not only has a high prevalence of 26.3% in the elderly [1], but its prevalence among children is also noteworthy. Fifteen years ago, the prevalence of FC in children ranged from 0.7% to 29.6% (median 12%) [2]. Seven years ago, a study reported a global overall prevalence of FC in children of 9.5%, with no association to gender [3]. Currently, another study indicates a prevalence of 8.17% for FC in European children < 4 years old, and 11.39% for FC in European children aged 4–18 years [4]. The causes of FC in children may involve factors such as diet and lifestyle, psychological aspects [5,6], and genetics [7,8]. It had been reported that childhood functional constipation has become an emerging public health problem [9].

Some systematic reviews have indicated that pharmacological treatments for children with FC include polyethylene glycol, lactulose, magnesium oxide or magnesium hydroxide, liquid paraffin, and probiotics. However, the collective findings suggest that polyethylene glycol is probably more effective than placebo and key comparator therapies and should be considered the standard of first-line care [10,11,12].

Shenqu Xiaoshi Oral Liquid, a distinctive traditional Chinese medicinal formulation designed to regulate and treat spleen and stomach disorders, was approved in China in 2014. Its primary components include charred Shenqu (*Massa Medicata Fermentata*), charred hawthorn (*Crataegi Fructus*), charred malt (*Hordei Fructus Germinatus*), *Paeoniae Radix Alba*, *Codonopsis Radix*, *Poria*, bran-fried *Atractylodis Macrocephalae Rhizoma*, *Aucklandiae Radix*, *Amomi Fructus*, vinegar-processed *Corydalis Rhizoma*, and honey-fried *Glycyrrhizae Radix* et *Rhizoma*, among others [13]. This formulation functions to promote digestion and regulate gastrointestinal motility. It is primarily used in clinical practice for the treatment of pediatric digestive conditions such as anorexia [14], functional dyspepsia [15], gut microbiota dysbiosis [16], and functional abdominal pain [17].

Recently, multiple clinical studies have indicated that Shenqu Xiaoshi Oral Liquid demonstrates unique advantages of traditional Chinese medicine in treating FC in children. This systematic review and meta-analysis aims to identify, evaluate, and synthesize data from randomized controlled trials or non-randomized controlled trials comparing Shenqu Xiaoshi Oral Liquid with control groups, specifically assessing its clinical efficacy and safety in children with functional constipation through key outcome measures.

## 2. Materials and Methods

This meta-analysis followed the Preferred Reporting Items for Systematic Reviews and Meta-Analyses (PRISMA) guideline 2020 [18]. The protocol for this study has been registered at the International Prospective Register of Systematic Reviews (PROSPERO) under the registration number (CRD420251243022).

### 2.1. Literature Search

A systematic review of electronic databases (PubMed, Embase, Scopus, Web of Science, Cochrane library, Chinese VIP Information Database, China National Knowledge Infrastructure (CNKI) and Wan Fang Med Database) was performed independently by two authors from inception to 20 October 2025. Meanwhile, we conducted an expanded reference search of the selected studies [19]. Search terms included “Shenqu Xiaoshi Oral Liquid” “Functional Constipation” “children” “child” “pediatrics”. The detailed search strategy is provided in Appendix A.

### 2.2. Selection of Studies

Inclusion criteria: (1) Study type: randomized controlled trials, retrospective studies and prospective studies; (2) Type of participants: pediatric patients under 18 years of age diagnosed with functional constipation, with the application of Rome IV criteria; (3) Type of intervention: intervention group used Shenqu Xiaoshi Oral Liquid, while control group did not; (4) Outcomes: the literature should provide accurate comprehensive statistical indicators: overall response rate; post-treatment stool passage interval; recurrence rate; post-recurrence stool passage interval; incidence of adverse events; motilin level and gastrin level.

Exclusion criteria: (1) duplicate publications, nursing reports, meta-analyses, conference papers, review papers, supplemental literature, letter to editor, etc.; (2) studies without sufficient data, control groups or interventions, clear methodology; (3) non-English or non-Chinese articles.

### 2.3. Data Extraction and Management

The inclusion criteria were pre-developed by all investigators. Two independent reviewers gradually eliminated the preliminary screening data, and made an independent decision on whether to include each paper according to the eligibility criteria. The data of the eligible studies were extracted separately using a pre-designed data extraction form, and if there was a disagreement on the final opinion of the literature, it was resolved by consensus with the senior authors. The following data were extracted: author’s name, research end time, study design, patients’ age, intervention groups and control groups, sample size, diagnostic criteria, treatment duration of Shenqu Xiaoshi Oral Liquid and outcomes. If there were differences in units of measurement between studies, they were uniformly converted to international unified units [19].

### 2.4. Quality Assessment

Trail quality was graded using the Cochrane risk of bias tool [20] for RCTs. Two reviewers assessed quality measurements for included studies, and discrepancies were adjudicated by collegial discussion. It comprised 7 items: random sequence generation, allocation concealment, blinding of participants and personnel, blinding of outcome assessment, incomplete outcome data, selective outcome reporting, and other bias. For each item, the risk of bias was assessed as “low risk” “unclear risk” or “high risk”. For the non-RCTs, the MINORS item [21] was used for methodological evaluation, with higher scores indicating stronger study design.

### 2.5. Statistical Analysis

All synthesis, calculation and analysis of extracted data were performed using Review Manager 5.3 and Stata 13.0 statistical software. The risk ratio (RR) and corresponding 95% confidence interval (95% CI) were calculated for dichotomous outcomes. Mean difference (MD) and 95% CI were calculated for continuous outcomes. Statistical heterogeneity was assessed using Cochran’s Q statistic, and Higgins and Thompson’s *I*^2^ (Tau^2^, Chi^2^ and *I*^2^ statistics). The fixed-effect model was used for meta-analysis when *p* ≥ 0.10 and *I*^2^ ≤ 50%, indicating adequate homogeneity. Otherwise, the random-effect model was used. Data of the outcomes were recorded for this meta-analysis when two or more trials reported the same outcome. Sensitivity analyses were performed to investigate the robustness of this meta-analysis. Meanwhile, the risk of publication bias was evaluated by funnel plot, Egger’s test and the trim-and-fill method. We aimed to assess potential publication bias based on the symmetry of the funnel plot. If it was difficult to judge whether the funnel plot was symmetric, Egger’s test was used to further clarify whether publication bias was present [22]. If the *p* value of Egger’s test was less than 0.05, we would further apply the trim-and-fill method to assess whether publication bias affected the robustness of the results. A *p* value < 0.05 was considered statistically significant.

## 3. Results

### 3.1. Results of the Literature Search

**Figure 1:**
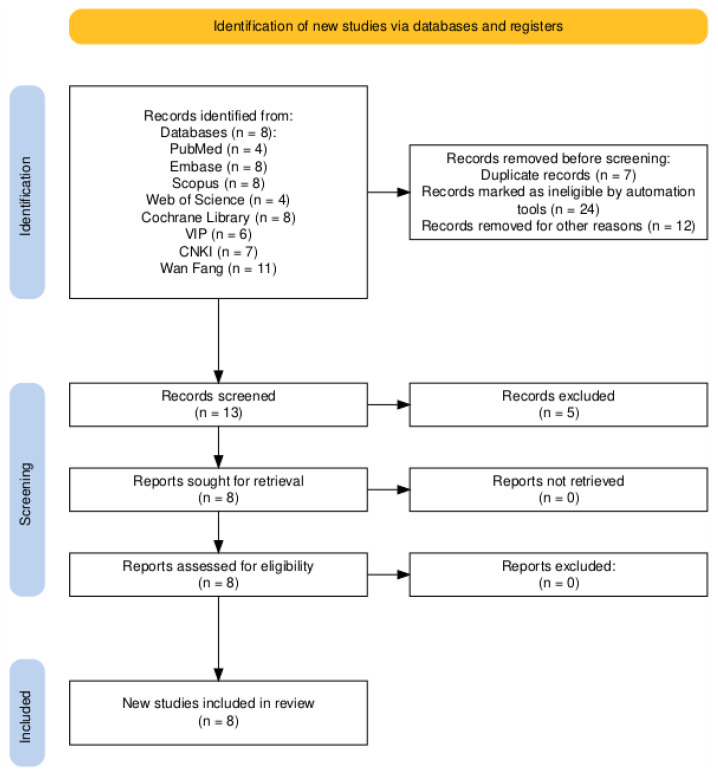
Preferred Reporting Items for Systematic Reviews and Meta-Analyses (PRISMA) flow chart.

Through a systematic search of eight databases: PubMed (n = 4), Embase (n = 8), Scopus (n = 8), Web of Science (n = 4), Cochrane Library (n = 8), VIP (n = 6), CNKI (n = 7), and Wan Fang (n = 11), we identified 56 records for initial screening. Full texts of all 56 articles were successfully retrieved. After careful review, 7 duplicate records and other irrelevant studies were excluded, resulting in 8 studies being included for analysis. Additionally, reference lists of the included studies were hand-searched to identify supplementary publications. Ultimately, 5 randomized controlled trials (RCTs) and 3 non-randomized studies involving 692 pediatric patients were included. The study selection process is detailed in the PRISMA flow diagram (Figure 1).

### 3.2. Characteristics of Selected Articles

All 8 included studies were conducted in China, involving a total of 692 pediatric patients, with 345 in the experimental group and 347 in the control group. Among the included studies, one study [23] used acupoint application and massage therapy in the control group, three studies [24,25,26] used probiotic therapy in the control group, and four studies [27,28,29,30] used lactulose therapy in the control group. All patients were diagnosed with functional constipation. All studies [23,24,25,26,27,28,29,30] explicitly stated that the Rome IV Criteria were used as the diagnostic standard. One study [25] administered Shenqu Xiaoshi Oral Liquid for 8 weeks, another study [28] administered it for 4 weeks, and the remaining studies used it for 2 weeks. All studies used Shenqu Xiaoshi Oral Liquid produced by the same manufacturer, and the oral administration method followed the same instructions. The main characteristics of the included studies are presented in Table 1.

### 3.3. Quality Assessment of the Included Studies

**Figure 2:**
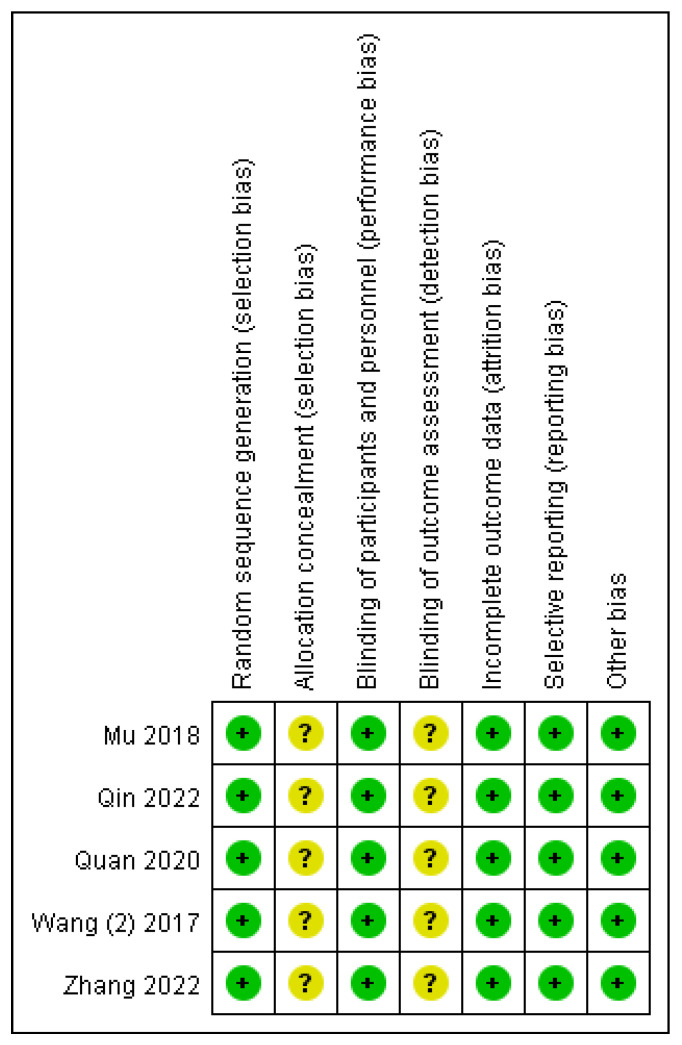
Risk of bias summary.

**Figure 3:**
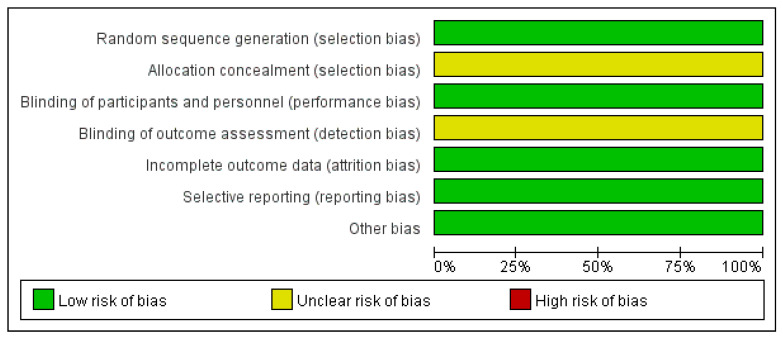
Risk of bias graph.

Five RCTs [24,25,26,27,30] were evaluated using the Cochrance Manual 5.1.0. The five RCTs all explicitly used a random number table method for random sequence generation, which is considered low risk. However, none of them described whether allocation concealment was implemented, which may pose a risk. Regarding blinding of participants and personnel, blinding was applied to both subjects and researchers, indicating low risk. As for blinding of outcome assessment, none of the studies described whether blinding was implemented, which may also present a risk. All five studies had complete data, no selective reporting, and no other biases (shown in Figure 2 and Figure 3). Due to the lack of clear descriptions regarding allocation concealment and blinding of outcome assessment in the included studies, the five RCTs carry some risk of bias. We must acknowledge that, coupled with the small sample sizes and the limited number of included studies, this may result in a reduction in the quality of evidence for our subsequent pooled analyses. For the remaining three non-randomized studies [23,28,29], the MINORS scale was used for quality assessment. This instrument consists of 12 items, with each item scoring a maximum of 2 points, yielding a total possible score of 24. Higher scores indicate better methodological quality. All three non-randomized studies received a total MINORS score of 17 or higher (shown in Table 2), demonstrating the good quality. We should be clearly aware that even if the included studies are all acceptable, publication bias may still exist. This is because the general publication process may overly pursue positive results, while some studies with negative results may remain unreported. Therefore, pooled analyses of high-quality studies may not necessarily yield reliable evidence.

### 3.4. Meta-Analysis of Outcomes

#### 3.4.1. Overall Response Rate

**Figure 4:**
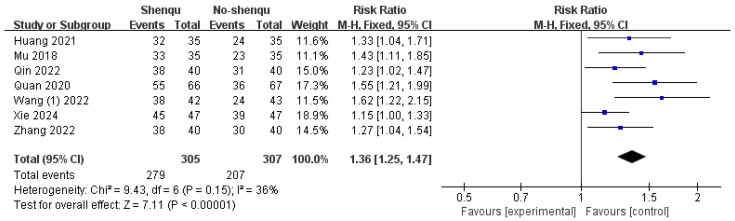
Forest plot of overall response rate.

Seven studies reported and compared the overall effective rates between children taking Shenqu Xiaoshi Oral Liquid and the control group. If the child’s defecation interval is less than 2 days, defecation frequency increases, defecation difficulty decreases, and the stool form is classified as type 4–6 according to the Bristol Stool Form Scale, it is considered effective. Cases that do not meet the above criteria are considered ineffective. A fixed-effects model was used (*I*^2^ = 36%, *p* = 0.15), and the results demonstrated that the combination of conventional treatment with Shenqu Xiaoshi Oral Liquid effectively alleviated constipation in children [RR = 1.36, 95% CI: (1.25, 1.47); z = 7.11, *p* < 0.00001]. Shown in Figure 4.

#### 3.4.2. Post-Treatment Stool Passage Interval

**Figure 5:**
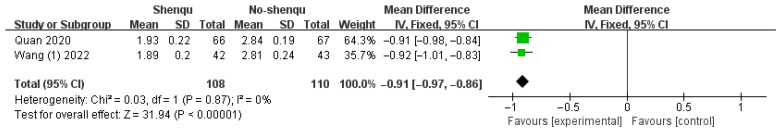
Forest plot of post-treatment stool passage interval.

Two studies reported and compared the interval between defecations in children after treatment with Shenqu Xiaoshi Oral Liquid. A fixed-effects model was used (*I*^2^ = 0%, *p* = 0.87), and the results showed that the combination of conventional treatment with Shenqu Xiaoshi Oral Liquid significantly reduced the interval between defecations [WMD = −0.91, 95% CI: (−0.97, −0.86); z = 31.94, *p* < 0.00001]. Shown in Figure 5.

#### 3.4.3. Recurrence Rate

**Figure 6:**
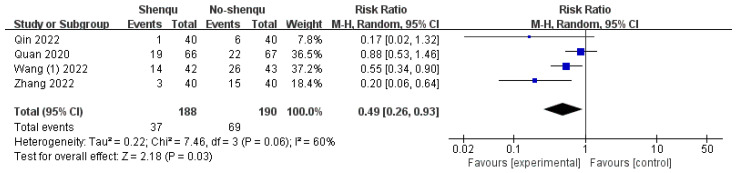
Forest plot of recurrence rate.

Four studies reported the recurrence rates of constipation in children using Shenqu Xiaoshi Oral Liquid versus control groups. Due to significant heterogeneity (*I*^2^ = 60%, *p* = 0.06), a random-effects model was applied. The results indicated that the recurrence rate was lower in the group receiving Shenqu Xiaoshi Oral Liquid compared to the control group [RR = 0.49, 95% CI: (0.26, 0.93); z = 2.18, *p* = 0.03]. Shown in Figure 6.

#### 3.4.4. Post-Recurrence Stool Passage Interval

**Figure 7:**
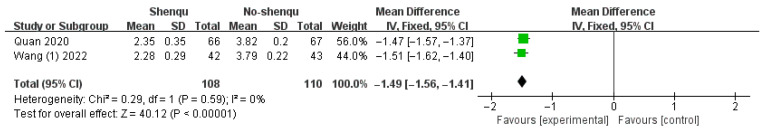
Forest plot of post-recurrence stool passage interval.

Two included studies reported the interval between defecations in children after recurrence of constipation following treatment with Shenqu Xiaoshi Oral Liquid. A fixed-effects model was employed for analysis (*I*^2^ = 0%, *p* = 0.59). The results demonstrated that, even after recurrence, the defecation interval in children who had received Shenqu Xiaoshi Oral Liquid was significantly shorter than that in the control group [WMD = −1.49, 95% CI: (−1.56, −1.41); z = 40.12, *p* < 0.00001]. Shown in Figure 7**.**

#### 3.4.5. Incidence of Adverse Events

**Figure 8:**
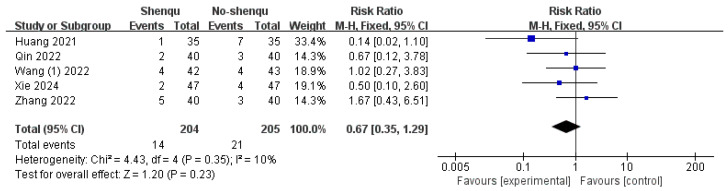
Forest plot of incidence of adverse events.

Five studies reported the incidence of adverse reactions after administration of Shenqu Xiaoshi Oral Liquid. Due to low heterogeneity, a fixed-effects model was applied (*I*^2^ = 10%, *p* = 0.35). The results showed no statistically significant difference between the Shenqu Xiaoshi Oral Liquid group and the control group, indicating that Shenqu Xiaoshi Oral Liquid did not increase the incidence of adverse reactions after treatment [RR = 0.67, 95% CI: (0.35, 1.29); z = 1.20, *p* = 0.23]. Shown in Figure 8**.**

#### 3.4.6. Motilin Level

**Figure 9:**
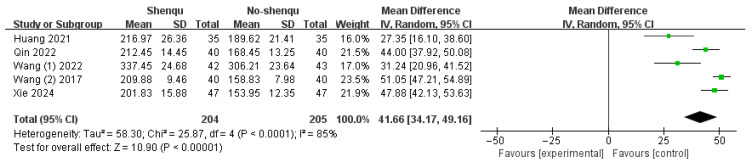
Forest plot of motilin level.

Five studies reported plasma motilin levels after treatment with Shenqu Xiaoshi Oral Liquid. Prior to treatment, there was no statistically significant difference in motilin levels between the Shenqu Xiaoshi Oral Liquid group and the control group across the five studies. Due to considerable heterogeneity (*I*^2^ = 85%, *p* < 0.0001), a random-effects model was employed. The results indicated that plasma motilin levels in children treated with Shenqu Xiaoshi Oral Liquid were markedly higher than those in the control group [WMD = 41.66, 95% CI: (34.17, 49.16); z = 10.90, *p* < 0.00001]. Shown in Figure 9**.**

### 3.5. Gastrin Level

**Figure 10:**
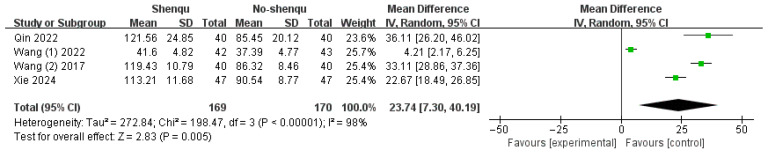
Forest plot of gastrin level.

Four studies reported plasma gastrin levels in children after treatment with Shenqu Xiaoshi Oral Liquid. Before treatment, there was no statistically significant difference in gastrin levels between the Shenqu Xiaoshi Oral Liquid group and the control group across the four studies. Due to considerable heterogeneity (*I*^2^ = 98%, *p* < 0.00001), a random-effects model was adopted. The results demonstrated that plasma gastrin levels in children treated with Shenqu Xiaoshi Oral Liquid were markedly higher than those in the control group [WMD = 23.74, 95% CI: (7.30, 40.19); z = 2.83, *p* = 0.005]. Shown in Figure 10**.**

### 3.6. Sensitivity Analysis

Furtherly, sensitivity analysis was performed to investigate the robustness of this meta-analysis. For the sensitivity analysis of these seven outcomes, we used the method of excluding included studies one by one—that is, we sequentially removed each study and recalculated the pooled results. The horizontal axis (Effect estimate) represents the recalculated pooled effect value after removing one study each time; the yellow dot (Estimate) represents the pooled effect after excluding that particular study; the short horizontal lines (CI limits) correspond to the 95% confidence intervals; and the three vertical lines (overall effect lines) represent the overall pooled result based on all studies.

Among these seven outcome indicators, all yellow dots were closely aligned with the overall effect lines, the width of the confidence intervals did not change substantially, and none crossed the null line, indicating that after removing any single study, the overall effect remained almost unchanged. That is, for the seven outcomes, the results of sensitivity analysis showed that the pooled results did not change significantly after the individual studies were excluded one by one, indicating that the results were relatively stable. All the results of the sensitivity analysis are shown in Appendix B.

### 3.7. Potential Biases in the Review Process

For all seven outcome measures, funnel plots were constructed (shown in Appendix C). However, it was difficult to visually assess publication bias directly from the funnel plots. Therefore, we further performed Egger’s test for outcomes that included two or more studies (shown in Table 3). The results showed that the *p*-values for total effective rate, incidence of adverse reactions, and motilin level were all less than 0.05. Accordingly, we applied the trim-and-fill method to these three outcomes.

**Figure 11:**
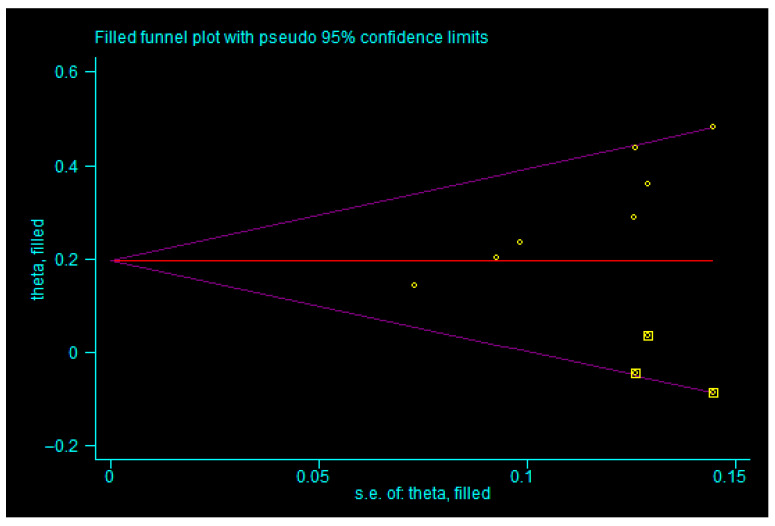
Trim-and-fill analysis of overall response rate. Note: yellow squares represent hypothetical studies added to achieve symmetry in the funnel plot; yellow dots represent actual studies. Even with the addition of three hypothetical studies, the results remain unaffected, indicating that publication bias does not compromise the robustness of our outcomes.

**Figure 12:**
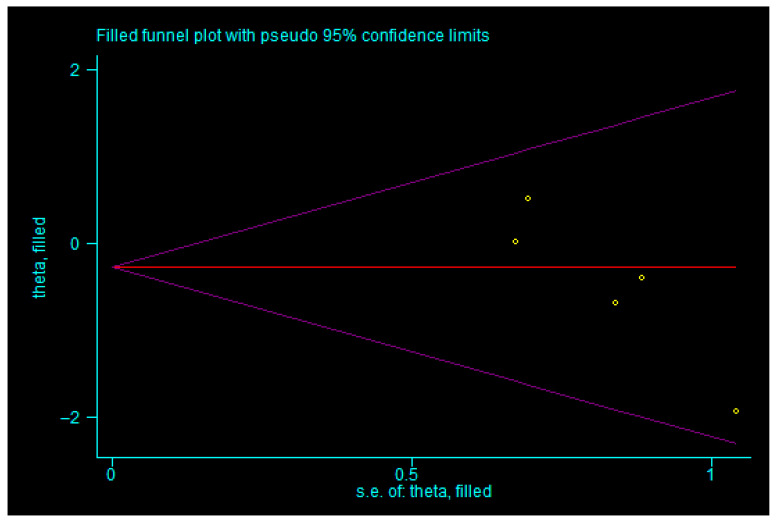
Trim-and-fill analysis of incidence of adverse events. Note: yellow squares represent hypothetical studies added to achieve funnel plot symmetry; yellow dots represent actual studies. In this plot, zero hypothetical studies were added, indicating that publication bias does not compromise the robustness of our results.

**Figure 13:**
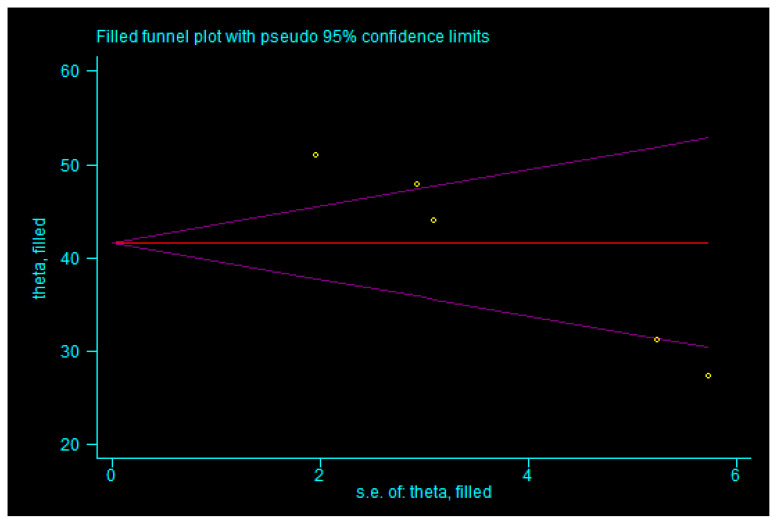
Trim-and-fill analysis of motilin level. Note: yellow squares represent hypothetical studies added to achieve funnel plot symmetry; yellow dots represent actual studies. In this plot, zero hypothetical studies were added, indicating that publication bias does not compromise the robustness of our results.

For the total effective rate, the trim-and-fill analysis indicated that three studies were imputed. After incorporating three additional studies (shown in Figure 11), the combined effect size remained statistically significant (*p* < 0.05), and the result was similar to the original estimate without reversal, suggesting that publication bias did not significantly influence the combined effect size and that the result was robust. For the incidence of adverse reactions, the trim-and-fill method imputed zero studies (shown in Figure 12), indicating that publication bias had no significant impact on the pooled effect size, which was therefore considered robust. Similarly, for motilin level, zero studies were imputed (shown in Figure 13), again suggesting that the pooled result was not substantially affected by publication bias and remained robust. These findings indicate that although there was evidence of publication bias for total effective rate, incidence of adverse reactions, and motilin level, such bias did not undermine the robustness of the meta-analysis results, which can still be considered reliable.

However, we also clearly recognize that the outcomes of recurrence rate, motilin, and gastrin exhibited high heterogeneity. Some researchers have argued that data for these outcomes should not be pooled. We attempted to explore sources of heterogeneity by performing subgroup analysis based on different control interventions, but this did not yield satisfactory explanations, and substantial heterogeneity remained unresolved. In addition, the number of patients in specific pediatric age subgroups was relatively small, making it difficult to conduct further subgroup analyses by age, region, gender, or treatment duration. Therefore, we consider that these results may still be reasonable from a clinical perspective. Based on our analysis and discussion, the observed heterogeneity may be attributed to the lack of standardized clinical guidelines for Shenqu Xiaoshi Oral Liquid, insufficient clinical experience among practitioners in its application, and inconsistencies in medical resources across different hospitals.

## 4. Discussion

The diagnosis of functional constipation in children is primarily based on clinical symptoms, with the Rome IV criteria serving as the current standard. These criteria are stratified according to whether the child is under or over 4 years of age [31]. If a patient meets the Rome IV diagnostic criteria and symptoms cannot be attributed to an underlying organic cause, a detailed medical history and physical examination are generally sufficient to establish the diagnosis. However, as noted in a recent study [32], the lack of objective severity parameters and reliance on self-reported symptoms may impede early identification. The Cleveland Clinic Constipation Score has been shown to be a valid and reliable tool that not only aids in diagnosing functional constipation but may also help assess its severity, potentially offering pediatricians additional guidance in clinical practice.

Non-pharmacological management of functional constipation in children includes adequate dietary fiber intake, sufficient fluid consumption, and regular physical activity [33]. Regarding pharmacotherapy, polyethylene glycol is considered the first-line agent for both disimpaction and maintenance therapy [34,35]. Improving the palatability of PEG can enhance treatment adherence, making it an effective and safe option for managing pediatric functional constipation [36]. Beyond standard PEG therapy, several adjuvant treatments have shown promising clinical benefits when combined with laxatives. These include acupuncture [37], pelvic floor physical therapy [38], anal botulinum toxin injection [39], transcutaneous electrical nerve stimulation [40], and a cow’s milk exclusion diet [41]. In addition, Shenqu Xiaoshi Oral Liquid, a traditional Chinese herbal formula, has also demonstrated unique and promising clinical efficacy as an adjunctive therapy for functional constipation in children

Shenqu Xiaoshi Oral Liquid is characterized by a sweet taste with a slight bitterness, which is generally well accepted by most children. Its multiple active ingredients contribute to various pharmacological effects, including promoting digestive secretion, regulating gastrointestinal smooth muscle contraction, accelerating gastric emptying, enhancing intestinal propulsion, improving small intestine absorption, as well as exerting anti-inflammatory and analgesic actions [42,43,44,45,46,47].

Reflecting the principles of Traditional Chinese Medicine [48], the formula of Shenqu Xiaoshi Oral Liquid is composed of several herbal components. The sovereign herbs (Jun Yao), charred *Shenqu*, charred *Hawthorn*, and charred *Germinated Barley*, function to strengthen the spleen, improve appetite, and resolve food stagnation. The minister herbs (Chen Yao), including *Codonopsis Root*, *Poria*, *Atractylodes Macrocephala*, and *White Peony Root*, serve to tonify the middle energizer and boost qi, fortify the spleen and calm the mind, eliminate dampness, promote diuresis, and soothe the liver to alleviate pain. The assistant herbs (Zuo Yao), such as *Aucklandia Root* and *Vinegar-processed Corydalis Rhizome*, are included to regulate qi and relieve pain, while *Amomum Fruit* helps to mobilize qi, harmonize the middle energizer, and awaken the spleen and stomach. *Licorice Root*, as the envoy herb (Shi Yao), harmonizes the actions of all components in the prescription. Together, these herbs achieve the therapeutic aims of promoting digestion, strengthening the stomach, invigorating the spleen, and regulating qi, thereby demonstrating distinctive Traditional Chinese Medicine characteristics.

Previous meta-analyses have indicated that Shenqu Xiaoshi Oral Liquid exhibits favorable clinical efficacy and broad applicability in children with functional dyspepsia [48,49,50]. However, to date, no meta-analysis has been conducted internationally regarding its use specifically for functional constipation in the pediatric population. Thus, the present study represents the first comprehensive systematic review and meta-analysis evaluating the clinical effectiveness and safety of Shenqu Xiaoshi Oral Liquid for treating functional constipation in children. In addition, a review of the currently available literature revealed no reported studies on the use of this formulation in adults with functional constipation. Consequently, it is not feasible to compare its clinical effects between pediatric and adult populations at this time.

This study investigated the therapeutic effects of traditional Chinese medicine on functional constipation in children. Looking ahead, further exploration is needed to determine whether other more effective treatments for pediatric functional constipation can enter clinical trials, or whether combination therapies could be considered. Potential insights may be gained from gastrointestinal preparation before pediatric endoscopy or from treatment approaches for functional constipation in adults [19,51,52,53,54]. Much work remains to be performed in the future.

### 4.1. Summary of Results

Compared with the control group, Shenqu Xiaoshi Oral Liquid demonstrated a higher overall response rate in the treatment of functional constipation in children. It significantly shortened the post-treatment defecation interval and was associated with a lower recurrence rate. Even in cases of recurrence, the defecation interval after relapse was significantly shorter than that in the control group. The incidence of adverse drug reactions showed no statistically significant difference between the two groups. Moreover, serum levels of motilin and gastrin were higher in the Shenqu Xiaoshi Oral Liquid group. It is noteworthy that although publication bias was detected, further validation using the trim-and-fill method indicated that the robustness of the final results was not compromised. In addition, for the outcome indicators of recurrence rate, serum motilin, and gastrin levels, heterogeneity persisted even after applying the random-effects model. The source of this heterogeneity could not be identified through subgroup analysis or other methods; therefore, caution is warranted when interpreting the validity of these three outcomes.

A notable disadvantage of this meta-analysis is the lack of direct comparison between Shenqu Xiaoshi Oral Liquid and polyethylene glycol. Thus, it remains unclear whether Shenqu Xiaoshi Oral Liquid is superior to polyethylene glycol, or whether combination therapy yields better outcomes. The control interventions included lactulose, probiotics, and traditional Chinese massage. Based on the current meta-analysis, Shenqu Xiaoshi Oral Liquid in combination with these therapies appears to provide enhanced therapeutic benefits.

The observed publication bias may be attributed to the inclusion of predominantly positive-result RCTs and non-randomized studies, while negative results might have been unpublished. Alternatively, the significant efficacy of Shenqu Xiaoshi Oral Liquid may have led to a scarcity of negative findings. To clarify this, further investigation into the pharmacological mechanisms of Shenqu Xiaoshi Oral Liquid is warranted.

In our clinical experience, some parents prefer Traditional Chinese medicine (TCM), which may be influenced by its thousands of years of historical use in China and the deep-rooted acceptance of TCM concepts among the Chinese population. However, the applicability and acceptability of TCM in pediatric populations outside China require further clinical research.

This study represents an up-to-date systematic review evaluating Shenqu Xiaoshi Oral Liquid, a TCM preparation with Chinese characteristics, for the treatment of functional constipation in children. Although current evidence may not yet establish definitive clinical guidelines, our findings offer practical insights to optimize therapeutic decision-making. Particularly in the context of rising global incidence of pediatric functional constipation, it is clinically relevant to explore treatment options with distinctive Chinese characteristics to protect children’s health. It must be emphasized that, given the inherent limitations of the available studies, the conclusions drawn here should be interpreted with caution. Future multicenter studies with extended follow-up periods are necessary to validate these preliminary observations.

### 4.2. Limitations of the Study

This study has several limitations. First, the meta-analysis revealed significant heterogeneity, which may be attributed to clinical variations across the included studies, such as differences in constipation severity, clinician experience, and inconsistent definitions of outcomes. Second, the generalizability of the findings is constrained by the predominance of small-sample, single-center studies conducted within China, raising concerns about geographical bias and limiting validation across diverse populations. Additionally, in the five RCTs, due to the lack of clear descriptions regarding allocation concealment and blinding of outcome assessment, the included studies carry some risk of bias, which reduces the quality grade of our evidence. Although high-quality RCTs may still be subject to publication bias and it is unlikely to have influenced our results, we nevertheless require further high-quality RCTs in the future. Finally, this study did not include a randomized comparison between Shenqu Xiaoshi Oral Liquid and polyethylene glycol, and there were insufficient subgroup analyses based on age (toddlers and adolescents subgroups), duration of medication, and regional distribution. Consequently, the hierarchy of evidence remains suboptimal. These limitations underscore the need for internationally optimized protocols and multicenter registries for the pharmacological treatment of functional constipation in children, to better capture longitudinal outcomes across varied healthcare settings.

## 5. Conclusions

In summary, Shenqu Xiaoshi Oral Liquid demonstrates higher effectiveness and lower recurrence rates in the treatment of functional constipation in children. Both the post-treatment and post-recurrence defecation intervals were shorter in the treatment group compared to the control group, without an increase in the incidence of adverse reactions. Moreover, it elevates plasma levels of motilin and gastrin. However, due to the influence of small sample sizes and potential heterogeneity, the results should be interpreted with caution. These neutral outcomes require further investigation with expanded study cohorts to validate their clinical significance.

## Figures and Tables

**Table 1:** Characteristics of included studies.

References	Time	Study Design	Age (Year)	Group	Sample Size (n)	Diagnostic Criteria	Dosing Duration	Outcomes
Huang et al. [23]	2021	Non-RCT	3 to 14	Acupoint application and massage	35	FC (Rome IV Criteria)	2 weeks	①⑤⑥
				Shenqu Xiaoshi Oral Liquid combined with acupoint application and massage	35			
Mu et al. [24]	2018	RCT	1 to 4	Bifidobacteria quadruple live bacteria	35	FC (Rome IV Criteria)	2 weeks	①
				Shenqu Xiaoshi Oral Liquid combined with bifidobacteria quadruple live bacteria	35			
Qin et al. [25]	2022	RCT	1 to 11	Bifidobacterium triple live bacteria	40	FC (Rome IV Criteria)	8 weeks	①③⑤⑥⑦
				Shenqu Xiaoshi Oral Liquid combined with bifidobacterium triple live bacteria	40			
Quan et al. [27]	2020	RCT	0.75 to 9	Lactulose	67	FC (Rome IV Criteria)	2 weeks	①②③④
				Shenqu Xiaoshi Oral Liquid combined with lactulose	66			
Wang (1) et al. [28]	2022	Non-RCT	1 to 11	Lactulose	43	FC (Rome IV Criteria)	4 weeks	①②③④⑤⑥⑦
				Shenqu Xiaoshi Oral Liquid combined with lactulose	42			
Wang (2) et al. [26]	2017	RCT	4 to 6	Bifidobacterium triple live bacteria	40	FC (Rome IV Criteria)	2 weeks	⑥⑦
				Shenqu Xiaoshi Oral Liquid combined with bifidobacterium triple live bacteria	40			
Xie et al. [29]	2024	Non-RCT	1 to 9	Lactulose	47	FC (Rome IV Criteria)	2 weeks	①⑤⑥⑦
				Shenqu Xiaoshi Oral Liquid combined with lactulose	47			
Zhang et al. [30]	2022	RCT	0.5 to 5	Lactulose	40	FC (Rome IV Criteria)	2 weeks	①③⑤
				Shenqu Xiaoshi Oral Liquid combined with lactulose	40			

**Note:** FC: Functional Constipation. ① Overall response rate; ② Post-treatment stool passage interval; ③ Recurrence rate; ④ Post-recurrence stool passage interval; ⑤ Incidence of adverse events; ⑥ Motilin level; ⑦ Gastrin level.

**Table 2:** MINORS scale of three non-RCTs.

Study	Huang 2021	Wang (1) 2022	Xie 2024
A clearly stated aim	2	2	2
Inclusion of consecutive patients	2	2	1
Prospective collection of data	1	1	1
Endpoints appropriate to the aim of the study	2	2	2
Unbiased assessment of the study endpoint	1	1	1
Follow-up period appropriate to the aim of the study	1	2	1
Loss to follow up less than 5%	0	0	0
Prospective calculation of the study size	0	0	1
An adequate control group	2	2	2
Contemporary groups	2	2	2
Baseline equivalence of groups	2	2	2
Adequate statistical analyses	2	2	2

**Table 3:** Publication bias of seven outcomes by Egger’s test.

Outcome	Egger’s Test	
	T	*p*
Overall response rate	6.56	0.001
Post-treatment stool passage interval	-	-
Recurrence rate	−1.89	0.199 *
Post-recurrence stool passage interval	-	-
Incidence of adverse events	−3.93	0.029
Motilin level	−10.03	0.002
Gastrin level	2.25	0.153 *

Note: *: *p* ≥ 0.05, for the remaining three outcomes without *, the trim and fill method was further applied.

**Figure A1:**
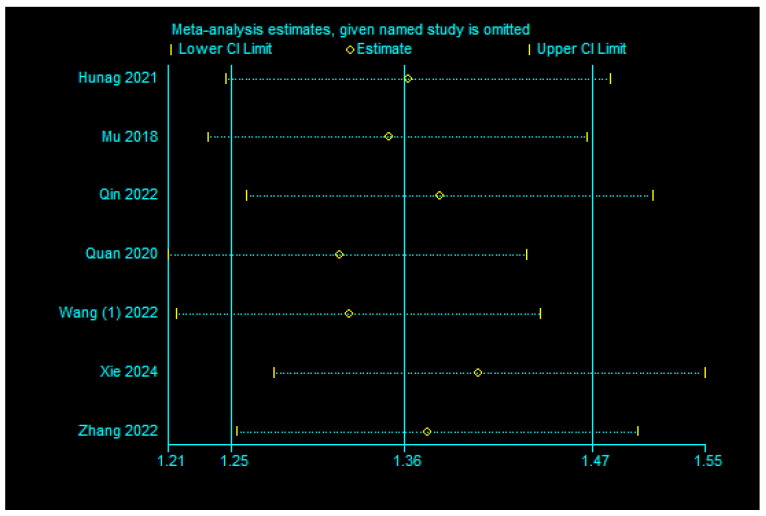
Sensitivity analysis of overall response rate.

**Figure A2:**
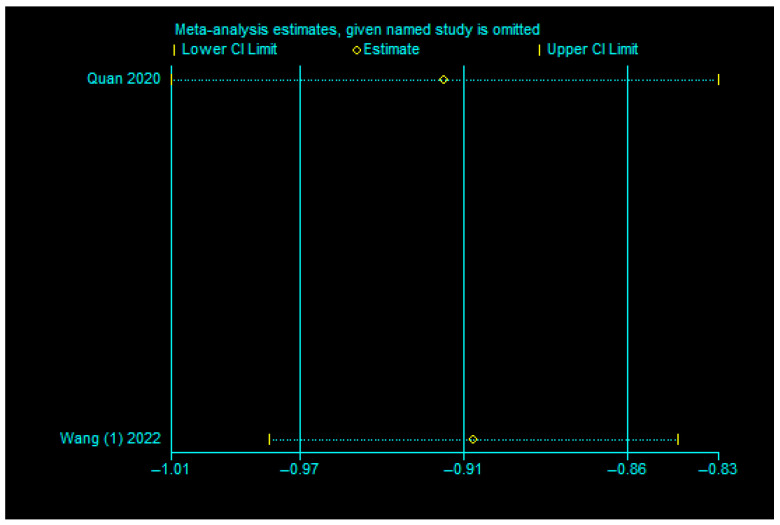
Sensitivity analysis of post-treatment stool passage interval.

**Figure A3:**
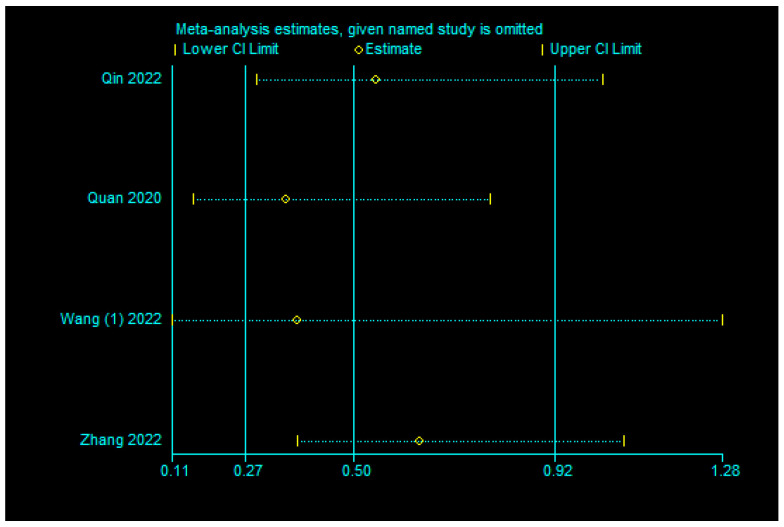
Sensitivity analysis of recurrence rate.

**Figure A4:**
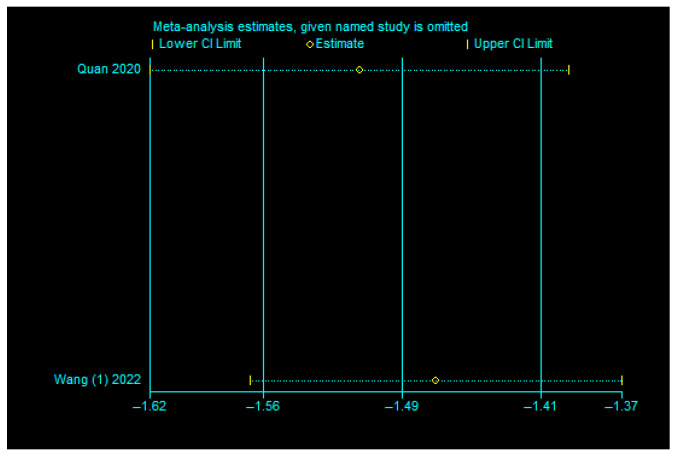
Sensitivity analysis of post-recurrence stool passage interval.

**Figure A5:**
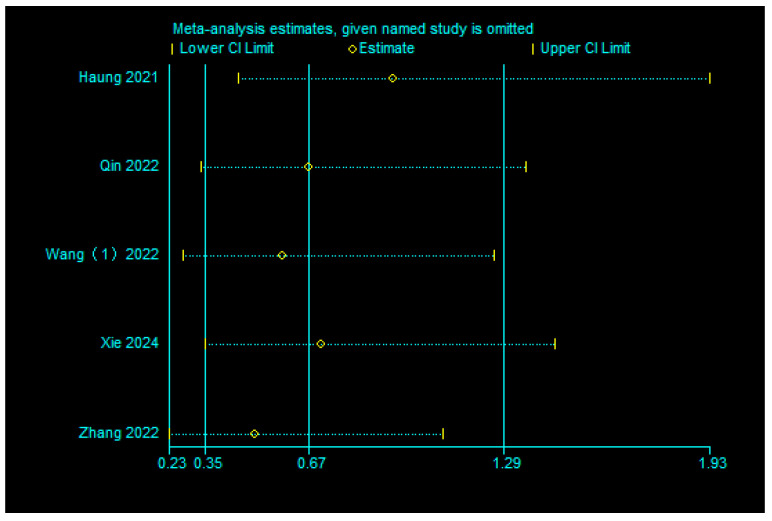
Sensitivity analysis of incidence of adverse events.

**Figure A6:**
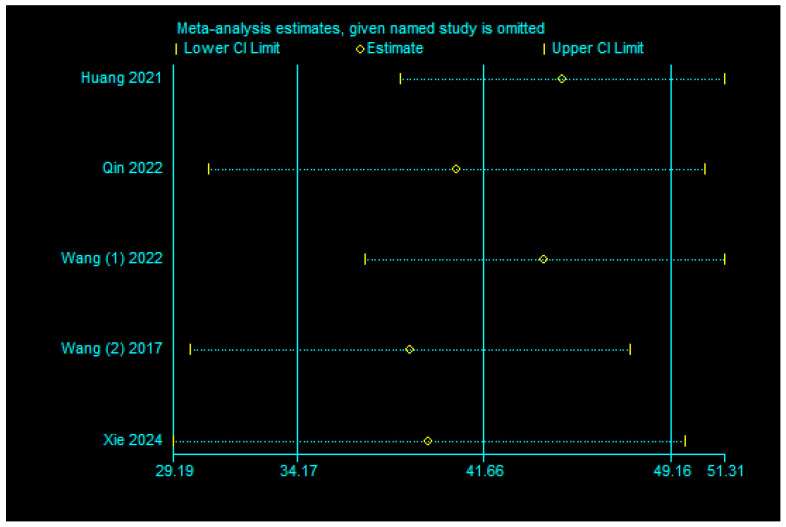
Sensitivity analysis of motilin level.

**Figure A7:**
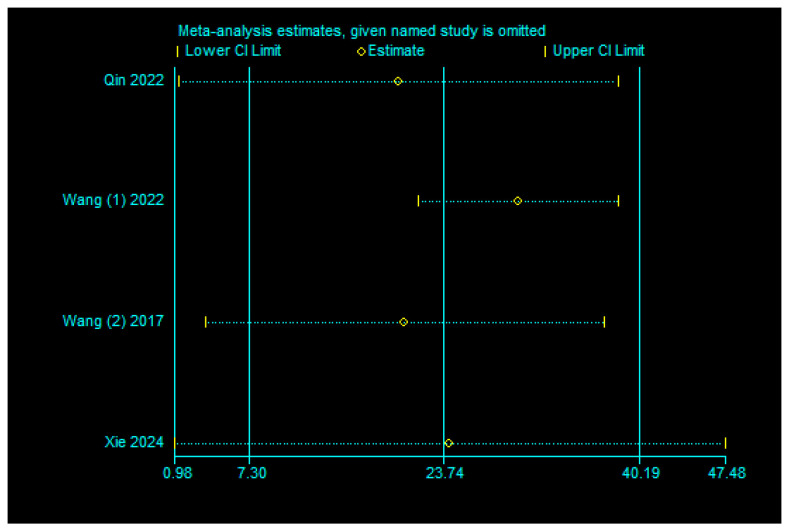
Sensitivity analysis of gastrin level.

**Figure A8:**
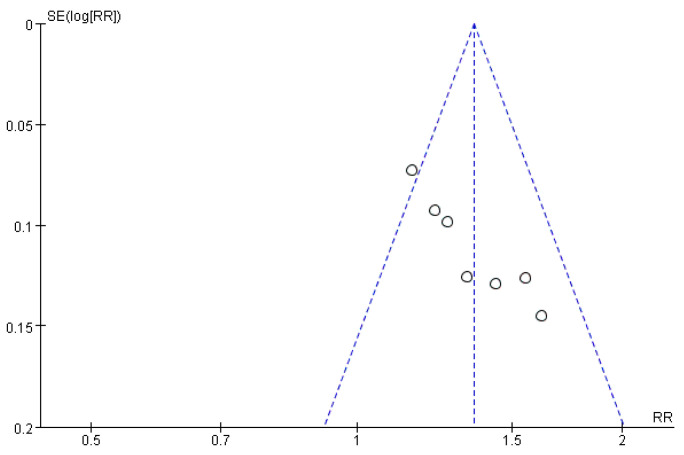
Funnel plot of overall response rate. Note: the asymmetric distribution of the funnel plot indicates publication bias.

**Figure A9:**
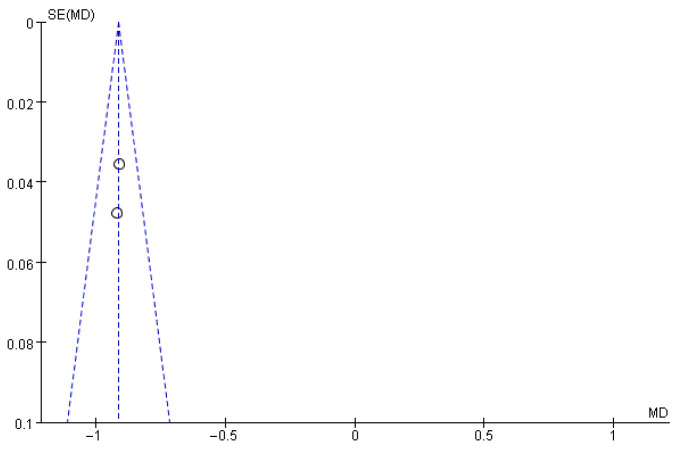
Funnel plot of post-treatment stool passage interval.

**Figure A10:**
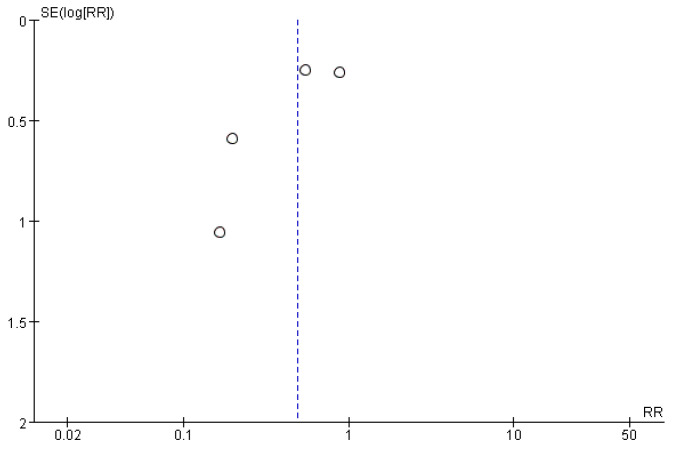
Funnel plot of recurrence rate.

**Figure A11:**
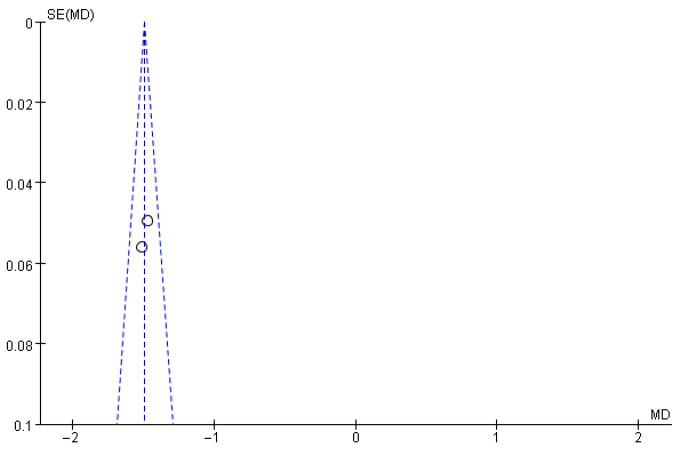
Funnel plot of post-recurrence stool passage interval.

**Figure A12:**
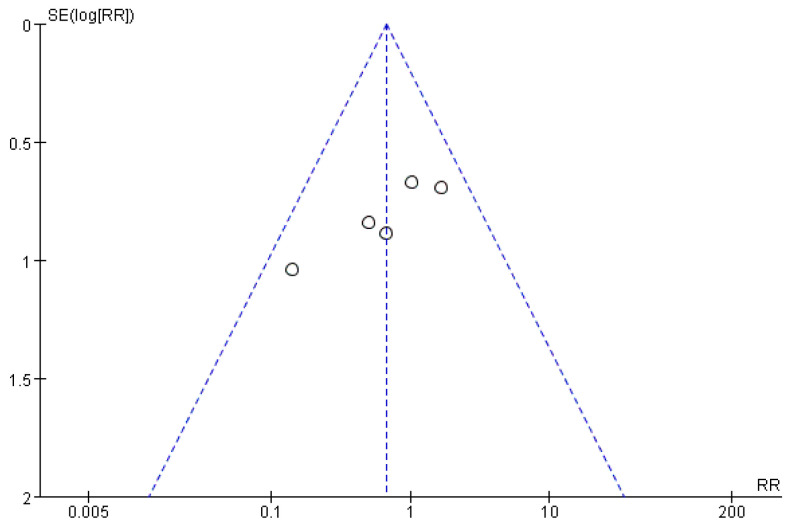
Funnel plot of incidence of adverse events. Note: the asymmetric distribution of the funnel plot indicates publication bias.

**Figure A13:**
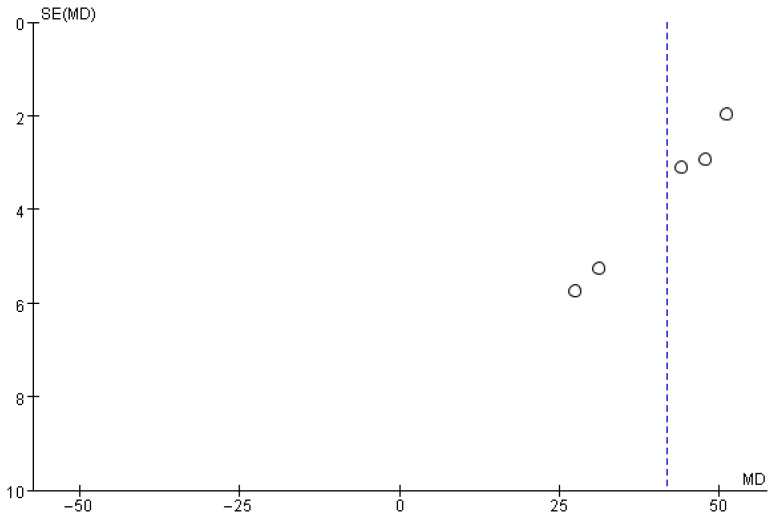
Funnel plot of motilin level. Note: the asymmetric distribution of the funnel plot indicates publication bias.

**Figure A14:**
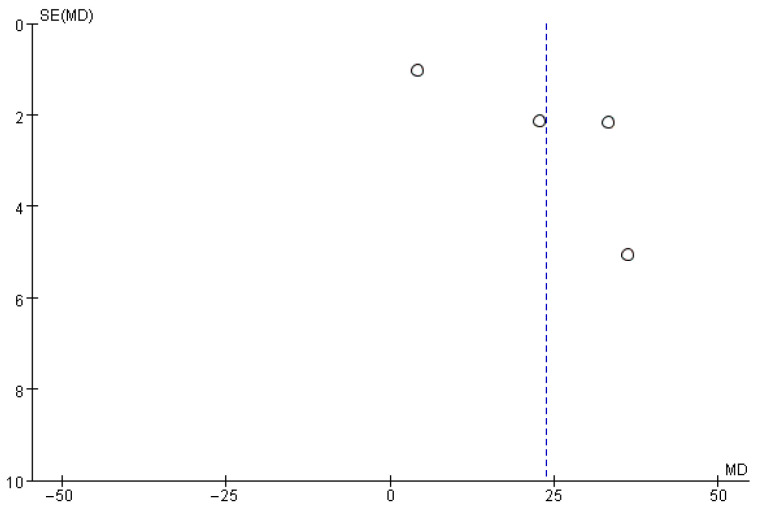
Funnel plot of gastrin level.

## Data Availability

No datasets were generated or analyzed during the current study.

## Appendix A. Search Strategy

**Table A1:** Search strategy used for the literature review. (From inception to 20 October 2025).

Databases	Search Details	Initial Number
PubMed	“Shenqu Xiaoshi Oral Liquid” [Title/Abstract]	4
Embase	‘Shenqu Xiaoshi Oral Liquid’ OR (Shenqu AND Xiaoshi AND Oral AND (‘Liquid’/exp OR Liquid))	8
Scopus	TITLE-ABS-KEY (Shenqu Xiaoshi Oral Liquid)	8
Web of Science	Shenqu Xiaoshi Oral Liquid (Topic)	4
Cochrane Library	(Shenqu Xiaoshi Oral Liquid): ti, ab, kw	8
VIP	高级检索： (任意字段 = 神曲消食口服液 AND (任意字段 = 便秘 OR 任意字段 = 功能性便秘))	6
CNKI	高级检索：（主题：神曲消食口服液） AND （主题：便秘 + 功能性便秘)	7
Wan Fang	高级检索：全部: (神曲消食口服液) and 全部: (便秘 OR 功能性便秘)	11
